# *APOE* Genotype in the Ethnic Majority and Minority Groups of Laos and the Implications for Non-Communicable Diseases

**DOI:** 10.1371/journal.pone.0155072

**Published:** 2016-05-11

**Authors:** Kaoru Midorikawa, Douangdao Soukaloun, Kongsap Akkhavong, Bouavanh Southivong, Oudayvone Rattanavong, Vikham Sengkhygnavong, Amphay Pyaluanglath, Saymongkhonh Sayasithsena, Satoshi Nakamura, Yutaka Midorikawa, Mariko Murata

**Affiliations:** 1 Department of Environmental and Molecular Medicine, Mie University Graduate School of Medicine, Tsu, Mie, Japan; 2 Mahosot Hospital, Ministry of Health, Vientiane, Lao PDR; 3 National Institute of Public Health, Ministry of Health, Vientiane, Lao PDR; 4 Department of Health Care, Ministry of Health, Vientiane, Lao PDR; 5 Laboratory of Epidemiology and Public Health, Graduate School of Nursing Science and Faculty of Nursing, Hiroshima Bunka Gakuen University, Kure, Hiroshima, Japan; 6 Department of Medical Nutrition, Faculty of Health, Suzuka University of Medical Science, Suzuka, Mie, Japan; Duke University, UNITED STATES

## Abstract

**Background:**

Increasing age is associated with elevated risk of non-communicable diseases, including dementia and Alzheimer’s disease (AD). The apolipoprotein E (*APOE*) ε4 allele is a risk factor not only for AD, but also for cognitive decline, depressive symptoms, stroke, hypertension, coronary heart disease, cardiovascular disease, and diabetes. The Lao People’s Democratic Republic (Laos) is undergoing development; consequently, life expectancy has risen. To evaluate the future risk of non-communicable diseases, we investigated *APOE* genotypes and anthropometric characteristics in the Laotian population.

**Methodology/Principal Findings:**

Subjects were 455 members of the Lao Loum majority and 354 members of ethnic minorities. *APOE* genotypes, anthropometric characteristics, blood pressure, and blood glucose were recorded. To compare individual changes, health examination data collected 5 years apart were obtained from a subset of Lao Loum subjects. *APOE* ε4 allele frequencies were higher among minorities (31.3%) than among Lao Loum (12.6%). In Lao Loum, but not in minorities, mean waist circumference and blood pressure increased significantly across age groups. Comparisons of health conditions between the beginning and end of the 5-year period revealed significant increases in obesity and blood glucose levels in Lao Loum. *APOE* ε4 carriers exhibited significant increases in resting heart rate in both ethnic groups.

**Conclusions/Significance:**

A higher ε4 allele frequency was observed in Laotian minorities than in the Laotian majority. Furthermore, higher obesity, blood pressure and blood glucose were observed in the middle-aged ethnic majority. Therefore, given these genetic and non-communicable disease risk factors, it seems likely that as the Laotian population ages, elevated rates of non-communicable aging-related diseases, such as dementia, will also become more prevalent.

## Introduction

As countries undergo economic development and associated lifestyle changes, the prevalence of non-communicable diseases such as diabetes, hypertension, cancer, cerebrovascular disease, cardiovascular disease, and neurodegenerative diseases may increase rapidly and become a serious problem, as in developed countries. The Lao People’s Democratic Republic (Laos) is undergoing a transition in the epidemiological structure of disease. Although the main causes of mortality and morbidity in Laos are still communicable diseases, including dengue fever, malaria, respiratory infections, and gastrointestinal diseases, the incidence of non-communicable diseases are increasing as the nation undergoes economic development[1]. The economy of Laos has grown steadily since the economic reforms of 1986, with GDP growth of around 8% over the last 5 years[1]. With the support of the World Health Organization (WHO) and other development partners, the rates of infectious diseases, especially malaria, have declined[1]. According to the World Alzheimer Report 2015, the global number of people living with dementia today is approximately 46.8 million; this number is expected to double every 20 years and reach 131.5 million in 2050[2]. Dementia, including Alzheimer’s disease (AD), is the most serious public health concern in developed countries. The same report predicts that the largest increase in dementia prevalence in the coming decades will occur in the low- and middle-income countries [2], including Laos. Over the past two decades, life expectancy in Laos rose by more than 10 years. In 2013, life expectancy at birth was 68 years for females and 65 years for males [3]. In the near future, as socioeconomic conditions continue to improve, the proportion of people 65 years or older will increase, and the number of patients with dementia, including AD, will grow.

Many studies have evaluated risk factors for dementia and AD. Potential risk factors include age, risk genes (*APOE*), lifestyle (diet, smoking, alcohol, and physical activity), and medical conditions (obesity, hypertension, stroke, diabetes, and hypercholesterolemia)[4–8]. Among these factors, the gene encoding apolipoprotein E gene (*APOE*) was identified as a major risk factor associated with the pathogenesis of AD[4, 9]. However, the contribution of *APOE* genotype to AD risk varies by ethnicity, sex, age and region[10–15]. Tang *et al*. reported that the *APOE* ε4 allele was less associated with the risk of AD in African Americans and Hispanics than in whites[15]. Additionally, the incidence of AD may be attributable to lifestyle and nutrition [4, 6, 16–20]. On the other hand, *APOE* ε4 is associated not only with AD but also with cognitive decline[21], depressive symptoms[22], stroke[23, 24], hypertension[25, 26], coronary heart disease[24, 26], cardiovascular disease[27, 28], and diabetes[29, 30]. In combination with environmental factors, *APOE* ε4 may influence the risk of non-communicable diseases.

In order to elucidate the clarify in risk of non-communicable diseases, including dementia and AD, between members of the Lao Loum majority and ethnic minorities, we investigated *APOE* ε4 frequency, anthropometric characteristics, blood pressure, and blood glucose. This is the first report to demonstrate the relationship between *APOE* genotypes and non-communicable diseases based on a comparison between the ethnic majority and minorities in Laos.

## Materials and Methods

### Study populations

We performed a survey in two urban villages, Phailom and Khoksa-ath in Vientiane Capital, and one rural village, Phuxay, in Attapeu Province in the south of the country during 2006–2013. The populations of the urban villages consisted primarily of the ethnic majority, Lao Loum, whereas the population of the rural village was largely Alak and Talieng ethnic minorities. The urban village of Phailom and Khoksa-ath, situated on the northern side of main national road number 6, are about 30 km and 25 km to the north of downtown Vientiane, respectively. The rural village of Phuxay lies about 40 km to the north of Attapeu city and is reachable by an unpaved road. Therefore, Phuxay does not have easy access to urban amenities, and economic conditions are poor, with little sanitation and no household electricity or running water at the time of the study. Phailom and Khoksa-ath do not have water supply (in 2014, some houses adjacent to the main street at Phailom village acquired water supply), but they do have household electricity and have been more affected by the recent changes in lifestyle.

### Subjects

The study subjects included 455 Lao Loum (150 males and 305 females) and 354 minorities (130 males and 224 females) during the surveillance period (Table 1). To determine the *APOE* genotype, we analyzed the blood of 439 of 455 Lao Loum and 345 of 354 minorities. For anthropometric characteristics, we targeted people over 20 years of age; 447 Lao Loum (143 males and 304 females) and 147 minorities (42 males and 105 females) were selected. Under the circumstances, it was difficult to recruit subjects aged 20 or older at the same rates in the urban villages of Vientiane Capital, because there were few elderly people in the minority village. Using the data of subjects over the age of 40 may be more relevant to age-related conditions. However the sample size of that is too small and the anthropometric and clinical characteristics changes from youth to aged people become unclear. So we used the data of subjects covering over the age of 20 to reveal the changes from youth to aged people clearly. For longitudinal analysis, we used the data collected from subjects who participated both at the start and end of the 5-year study period, a total of 74 Lao Loum (25 males and 49 females). We compared the changes in anthropometric and clinical characteristics at the start and end of the 5-year period. Of the minorities, only 12 subjects (eight of whom were under 20 years old; one male and three females were aged 20 or older) participated twice. Because of the small sample size, we did not analyze changes over time in minorities.

**Table 1:** Breakdown of participants by age and gender category.

	Male				Female			
	Lao Loum		Minorities		Lao Loum		Minorities	
Age group (years)	No.	(%)	No.	(%)	No.	(%)	No.	(%)
<20	7	(4.7)	88	(67.7)	1	(0.3)	119	(53.1)
20–29	5	(3.3)	10	(7.7)	17	(5.6)	34	(15.2)
30–39	22	(14.7)	12	(9.2)	54	(17.7)	32	(14.3)
40–49	27	(18.0)	13	(10.0)	71	(23.3)	15	(6.7)
50–59	50	(33.3)	3	(2.3)	91	(29.8)	14	(6.3)
60≤	39	(26.0)	4	(3.1)	71	(23.3)	10	(4.5)
Total	150	(100.0)	130	(100.0)	305	(100.0)	224	(100.0)

No.; Number.

### Ethics Approval

The project received ethics approval from Mie University, Japan (No.1208) and the Ministry of Health National Ethic Committee For Health Research, Laos (No.102). Written informed consent for the participation in the study was obtained from participants. The next of kin or guardians of the children and the minorities gave written informed consent on behalf of them. At the end of their examination, each participant was informed of his or her status with regard to risk for glucose intolerance, hypertension, and obesity. People with newly discovered diabetes or hypertension were referred for clinical appraisal and advised by the doctor.

### Procedures

Before each survey, we informed participants of the purpose and procedures of the study. The surveys were conducted at the village community center, the village temple or the primary school in the village site. Obesity, diabetes, and hypertension were classified according to the most recent WHO recommendations[31, 32]. Participants received medical advice from doctors.

Random blood glucose concentration was measured on site with a blood glucose analyzer (Dexter ZII or Breeze 2, Bayer Medical Co. Leverkusen, Germany). Hemoglobin A 1c (HbA1c) was detected by DCA 2000® Analyzer (Siemens, Tokyo, Japan) or A1CNow plus (Bayer Medical Co. Leverkusen, Germany). Blood pressure and resting heart rate (HR) were measured using a brachiohemopiezometer (EW3106, National Co. Tokyo, Japan), and body weight was measured using a digital weight scale (HD654, TANITA Co. Tokyo, Japan). Height was measured using a stadiometer (seca213, Seca yk, Chiba, Japan). The measurement of waist circumference (WC) was taken with a measuring tape (umfangmessband 203cm, Seka yk. Chiba, Japan) in a horizontal plane, midway between the inferior margin of the iliac crest, according to the International Diabetes Federation consensus worldwide definition of metabolic syndrome[33].

### Genotyping

For genetic analysis, whole blood from each subject was spotted on filter paper cards (FTA card or FTA Elute, Whatman, Maidstone, UK), which were air-dried for 2 h and individually collated in air-tight sealed bags for DNA analysis. Genomic DNA was extracted and purified from whole blood on filter paper cards in accordance with the manufacturer's instructions. Genotyping was performed in 96 well-plates; the final reaction volume was 10 μl including 5 ng of genomic DNA, 5 μl of TaqMan universal master mix II no UNG, and 0.5 μl of 20 x SNP genotyping assay mix (ID number C_904973_10 for rs7412 and C_3084793_20 for rs429358). PCR plates were read after heating at 95°C for 10 min, followed by 40 cycles of denaturation at 95°C for 15 s and annealing/extension at 60°C for 1 min. Single-nucleotide polymorphisms (SNPs) rs7412 and rs429358, which determine the *APOE* ε2, ε3 and ε4 alleles, were analyzed by TaqMan® polymerase chain reaction (PCR) assays (SNP assays-on-demand) on a StepOne analyzer using the StepOne software v2.3. All assays, machines, and software were from Applied Biosystems (Applied Biosystems, CA, USA). Blood samples collected in 2012 and 2013 were analyzed using TaqMan assays and a real-time PCR machine, whereas others were sent to Aoba Genetics Inc. (Yokohama, Japan) for genotype analysis.

### Statistical analysis

We analyzed the data with SPSS Statistics 20 software (IBM). Trends across age groups were compared by Jonkheere-Terpstra test. Comparisons between two study groups were analyzed by Mann-Whitney U test, or by chi-square test or Fisher’s exact test if the frequencies were small. The Wilcoxon signed-rank test was used to compare Lao Loum health examination data collected 5 years apart. A value of *P* < 0.05 was taken to be statistically significant. Simultaneous multiple regression models were used to evaluate the correlations between dependent variables (weight, height, BMI, WC, SBP, DBP, HR, blood glucose) and independent variables (age, sex, ethnicity, *APOE* ε4 carrier). In figures, box plots indicate the median, 25^th^, and 75^th^ percentiles. The range within 1.5 times the length of the interquartile range is shown. Outliers are represented by open circles (values that were between 1.5 and 3 times greater than the interquartile range) and by asterisks (more than 3 times greater).

## Results

### *APOE* genotypes

To our knowledge, this is the first study to investigate *APOE* genotypes of people in Laos. Table 2A shows that 59.7% of the Lao Loum and 40% of minorities carried the *APOE* ε3/ε3 genotypes. Although the ε3/ε3 genotype is the most common genotype in both ethnic groups, the proportions of genotypes in two groups were significantly different (*P* < 0.001). The prevalence of the *APOE* ε4/ε4 genotype and ε4 allele frequencies of minorities (9.9% and 31.3%) were higher than those of Lao Loum (0.5% and 12.6%). In addition, the proportions of genotypes were significantly different in each minority group, Talieng (*P* < 0.001) and Alak (*P* < 0.001), relative to Lao Loum. In the two minority groups, the *APOE* ε4/ε4 genotype frequencies for Talieng and Alak were 6.8% and 14.0%, respectively, and the *APOE* ε4 allele frequencies were 24.3% and 40.1%, respectively. There was a difference in age distribution between the majority and minority populations, and we selected subjects aged 20 and older for further analyses. The proportions of genotypes in subjects aged 20 and older (Table 2B) were almost identical to those in the overall subject population (Table 2A). In addition, the *APOE* ε4 carrier frequency was twice as high in minority groups (51.7%) than in Lao Loum (24.8%) (*P* < 0.001, Table 2C). To confirm the influence of sex and age on the genotype distribution, we used the chi-square test to analyze *APOE* ε4 carrier/non-carrier status as a function of sex and age demographics. For both ethnicities, neither sex nor age demographics significantly affected carrier status (data not shown).

**Table 2:** Genotype distribution of the *APOE* polymorphism in Laos.

**(A) *APOE* genotypes in Laos (all subjects)**								
	Majority		Minorities		Breakdown of minorities			
	Lao Loum		Talieng & Alak & others		Talieng		Alak	
Genotypes	No.	(%)	No.	(%)	No.	(%)	No.	(%)
ε2/ε2	9	(2.1)	0	(0.0)	0	(0.0)	0	(0.0)
ε2/ε3	59	(13.4)	25	(7.2)	13	(7.3)	12	(7.6)
ε2/ε4	19	(4.3)	27	(7.8)	6	(3.4)	20	(12.7)
ε3/ε3	262	(59.7)	138	(40.0)	90	(50.8)	41	(26.1)
ε3/ε4	88	(20.0)	121	(35.1)	56	(31.6)	62	(39.5)
ε4/ε4	2	(0.5)	34	(9.9)	12	(6.8)	22	(14.0)
Total	439	(100.0)	345	(100.0) ^1)^	177	(100.0) ^2)^	157	(100.0) ^3)^^,^ ^4)^
**(B) *APOE* genotypes in Laos (subjects aged 20 and older)**								
	Lao Loum		Talieng & Alak & others		Talieng		Alak	
Genotypes	No.	(%)	No.	(%)	No.	(%)	No.	(%)
ε2/ε2	9	(2.1)	0	(0.0)	0	(0.0)	0	(0.0)
ε2/ε3	59	(13.7)	18	(12.4)	8	(11.9)	10	(13.3)
ε2/ε4	19	(4.4)	11	(7.6)	2	(3.0)	8	(10.7)
ε3/ε3	256	(59.4)	52	(35.9)	32	(47.8)	18	(24.0)
ε3/ε4	86	(20.0)	52	(35.9)	19	(28.4)	33	(44.0)
ε4/ε4	2	(0.5)	12	(8.3)	6	(9.0)	6	(8.0)
Total	431	(100.0)	145	(100.0) ^1)^	67	(100.0) ^2)^	75	(100.0) ^3)^^,^ ^5)^
**(C) *APOE*** ε**4 groups in Laos (subjects aged 20 and older)**								
*APOE*ε4 allele Group		Lao Loum				Talieng & Alak & others		
		No.	(%)			No.	(%)		
ε4 Carrier		107	(24.8)			75	(51.7)	
Non-carrier		324	(75.2)			70	(48.3)	
Total		431	(100)			145	(100.0)^1)^	

Data are number (%) of subjects for genotypes and number (%) of chromosomes for alleles.

Comparison of Lao Loum with Minorities, chi-square test (^1)^*P*<0.001)

Comparison of Lao Loum with Talieng, chi-square test (^2)^*P*<0.001)

Comparison of Lao Loum with Alak, chi-square test (^3)^*P*<0.001)

Comparison of Talieng with Alak, chi-square test (^4)^*P*<0.001, ^5)^ *P*<0.05).

### Comparison of anthropometric and clinical characteristics between Lao Loum and minorities

Trends for mean body weight, height, body-mass index (BMI), waist circumference (WC), systolic blood pressure (SBP), diastolic blood pressure (DBP), resting heart rate (HR), blood glucose and HbA1c across age groups are summarized in Table 3 and supporting information S1 Fig. BMI significantly increased among people in their 20s to 40s groups in Lao Loum females and seemed to be highest in middle age of both sexes, but significantly deceased and seemed to be lowest in middle age of minority females. In Lao Loum of both sexes, WC tended to increase significantly among people in their 20s to 40s and seemed to be highest in middle age, whereas WC decreased and seemed to be lowest in middle age among female minorities. Among both males and females of Lao Loum, although body weight tended to decrease significantly in all age groups, body weight seemed to be highest in middle age and then decreased for advanced age. On the other hands, among female minorities, body weight also tended to significantly decrease, but seemed to be lowest in middle age as opposed to Lao Loum. Especially in their 40s group of minority females, body weight, BMI, and WC seemed to be lowest as opposed to Lao Loum in middle age. An obese tendency in the middle age was seen in Lao Loum but not in minorities. The trend of height of Lao Loum of both sexes and female minorities tended to significantly decrease. This means height of younger groups was higher than that of elder groups. In Lao Loum of both sexes, but not in minorities, SBP and DBP tended to increase across age groups. Resting HR significantly decreased in females of both Lao Loum and minorities, although it showed an increase in their 20s to 40s of Lao Loum females. Blood glucose increased significantly within all Lao Loum age groups and minority male in their 20s to 40s. HbA1c in Lao Loum females significantly increased over the course of aging from the 20s to the 40s.

**Table 3:** Anthropometric and clinical characteristics of age categories in Lao Loum and minorities.

		Male						Female					
		Lao Loum			Minorities			Lao Loum			Minorities		
	Age group (years)	No.	mean±SD	*Trend test P*^1)^ [*P*^2)^]	No.	mean±SD	*Trend test P*^1)^ [*P*^2)^]	No.	mean±SD	*Trend test P*^1)^ [*P*^2)^]	No.	mean±SD	*Trend test P*^1)^ [*P*^2)^]
Weight	20–29	5	57.0 ± 5.1	**0.001**	10	49.7 ± 5.7	0.275	17	52.7 ± 13.1	**<0.001**	34	43.9 ± 5.6	**0.036**
(kg)	30–39	22	65.5 ± 7.4	[0.473]	12	52.6 ± 5.6	[0.332]	54	59.3 ± 11.3	[0.088]	32	42.6 ± 7.7	**[0.004]**
	40–49	27	64.7 ± 9.0		13	47.5 ± 4.3		71	59.1 ± 10.5		15	38.2 ± 4.2	
	50–59	50	58.3 ± 9.6		3	54.3 ±5.5		91	56.3 ± 11.0		14	42.3 ± 7.6	
	60≤	39	58.3 ± 13.6		4	44.8 ± 5.0		70	50.7 ± 8.1		10	42.6 ± 7.7	
Height	20–29	5	163.5 ± 7.5	**0.001**	10	155.5 ± 5.2	0.928	16	154.8 ± 2.9	**<0.001**	34	148.8 ± 5.8	0.838
(cm)	30–39	22	164.3 ± 6.4	[0.286]	12	159.5 ± 3.9	[0.952]	54	153.8 ± 5.1	**[0.001]**	32	147.9 ± 5.4	**[0.018]**
	40–49	27	161.6 ± 5.5		13	156.5 ± 4.4		71	151.1 ± 4.8		15	145.0 ± 2.8	
	50–59	50	160.9 ± 4.8		3	162.1 ± 1.7		91	151.0 ± 5.5		14	149.9 ± 6.0	
	60≤	39	158.7 ± 6.6		4	154.5 ± 3.9		70	148.3 ± 5.5		10	150.0 ± 7.3	
BMI	20–29	5	21.4 ± 2.2	0.071	10	20.6 ± 2.8	0.212	16	22.3 ± 5.3	0.058	34	19.9 ± 2.9	**0.021**
	30–39	22	24.3 ± 2.8	[0.131]	12	20.7 ± 2.1	[0.275]	54	25.1 ± 4.6	**[0.005]**	32	19.4 ± 2.7	**[0.030]**
	40–49	27	24.8 ± 3.5		13	19.4 ± 1.2		71	25.8 ± 4.2		15	18.2 ± 2.1	
	50–59	50	22.5 ± 3.3		3	20.6 ± 1.7		91	24.6 ± 4.1		14	18.8 ± 2.9	
	60≤	39	23.0 ± 4.4		4	18.8 ± 2.8		70	23.0 ± 3.2		10	18.9 ± 2.7	
WC	20–29	5	75.1 ± 4.7	0.784	3	66.8 ± 1.4	0.159	17	77.2 ± 11.2	0.12	20	67.3 ± 4.9	0.364
(cm)	30–39	22	83.3 ± 6.6	**[0.003]**	7	73.3 ± 4.7	[0.893]	54	82.9 ± 10.3	**[0.001]**	16	65.3 ± 6.0	**[0.002]**
	40–49	27	87.3 ± 8.9		7	69.8 ± 2.5		71	86.2 ± 10.4		8	61.2 ± 2.5	
	50–59	50	80.3 ± 9.3		3	76.4 ± 3.9		91	84.2 ± 11.1		9	67.2 ± 8.6	
	60≤	39	84.0 ± 13.9		2	75.1 ± 12.9		70	85.0 ± 9.9		8	70.1 ± 10.6	
SBP	20–29	5	110.7 ± 8.3	**0.001**	10	126.2 ± 21.8	0.980	17	103.4 ± 12.8	**< 0.001**	33	124.1 ± 10.1	0.105
(mmHg)	30–39	22	119.1 ± 12.9	**[0.049]**	12	124.6 ± 14.4	[0.934]	54	116.0 ± 14.1	**[< 0.001]**	29	130.6 ± 16.2	[0.156]
	40–49	27	123.0 ± 15.5		11	123.5 ± 12.1		71	121.6 ± 16.1		15	128.4 ± 12.4	
	50–59	50	124.1 ± 14.1		3	120.7 ± 14.6		91	126.5 ± 17.9		14	130.8 ± 16.5	
	60≤	39	130.0± 18.5		3	130.7 ± 25.4		71	134.7 ± 21.4		10	132.5 ± 18.6	
DBP	20–29	5	69.4 ± 6.0	0.567	10	79.1 ± 8.0	0.810	17	68.4 ± 9.5	**0.004**	33	81.6 ± 10.0	0.740
(mmHg)	30–39	22	75.6 ± 8.9	**[0.009]**	12	81.1 ± 8.2	[0.715]	54	74.6 ± 10.8	**[0.001]**	29	87.4 ± 14.6	[0.808]
	40–49	27	79.1 ± 8.8		11	79.6 ± 5.9		71	77.3 ± 9.1		15	78.5 ± 9.1	
	50–59	50	78.4 ± 9.1		3	75.3 ± 8.4		91	78.6 ± 9.2		14	81.6 ± 7.9	
	60≤	39	76.6 ± 8.7		3	78.7 ± 1.2		71	76.9 ± 10.0		10	84.1 ± 12.2	
HR	20–29	5	78.2 ± 18.3	0.712	10	71.3 ± 13.8	0.181	17	83.5 ± 17.4	**0.040**	33	89.2 ± 14.4	**0.029**
(bpm)	30–39	22	78.3 ± 11.7	[0.652]	12	68.4 ± 8.2	[0.124]	54	80.1 ± 10.1	**[0.031]**	29	78.5 ± 10.4	**[0.002]**
	40–49	27	75.4 ± 11.0		11	74.0 ± 6.2		71	84.0 ± 10.6		15	78.5 ± 7.3	
	50–59	50	75.0 ± 13.1		3	68.7 ± 7.8		91	79.1 ± 13.7		14	82.4 ± 13.6	
	60≤	39	78.9 ± 13.3		3	74.0 ± 2.7		71	78.1 ± 9.5		10	81.7 ± 10.1	
Blood	20–29	5	103.4 ± 18.2	**0.006**	10	105.6 ± 20.4	0.066	15	95.3 ± 15.2	**<0.001**	34	105.3 ± 21.9	0.195
glucose	30–39	22	122.5 ± 53.2	[0.179]	12	116.2 ± 24.6	**[0.044]**	47	113.2 ± 39.4	[0.099]	32	100.3 ± 24.8	[0.129]
(mg/dL)	40–49	27	150.3±100.7		13	119.8 ± 17.4		67	127.4 ± 73.5		15	96.1 ± 14.2	
	50–59	49	140.7 ± 77.1		3	115.0 ± 28.9		91	122.6 ± 51.8		14	112.1 ± 27.9	
	60≤	38	168.0±106.9		4	120.5 ± 22.6		69	133.8 ± 66.5		10	130.1 ± 29.6	
HbA1c	20–29	3	5.0 ± 0.9	0.095		ND		6	4.9 ± 0.5	0.316		ND	
(%)	30–39	19	5.7 ± 1.5	[0.081]		ND		39	5.6 ± 1.0	**[0.032]**		ND	
	40–49	19	6.5 ± 2.1			ND		44	6.0 ± 1.8			ND	
	50–59	30	6.0 ± 1.9			ND		44	5.8 ± 0.9			ND	
	60≤	25	6.8 ± 2.2			ND		41	5.8 ± 1.4			ND	

ND: No data, Significant *P* values (<0.05) are in bold,

1) Jonkheere-Terpstra test in age groups 20–29, 30–39, 40–49, 50–59, and ≥60 years

2) Jonkheere-Terpstra test in age groups 20–29, 30–39, and 40–49 years

WC; Waist circumference, SBP; Systolic blood pressure, DBP; Diastolic blood pressure, HR; Heart rate.

### Changes in Lao Loum anthropometric data collected 5 years apart

Table 4 shows a comparison of anthropometric and clinical characteristics data collected at the beginning and end of a 5-year period from Lao Loum subjects. Body weight, BMI, WC increased significantly in males (*P* = 0.010, 0.009, 0.037, respectively) and females (*P* < 0.001, < 0.001, < 0.001, respectively). Height, SBP, and DBP remained similar in both males and females. Resting HR increased significantly in males (*P* = 0.049) and trended toward an increase in females (*P* = 0.056). Blood glucose also significantly increased in both males (*P* = 0.044) and females (*P* = 0.002). HbA1c increased only in females (*P* < 0.001), but not in males (*P* = 0.172).

**Table 4:** A comparison of Lao Loum anthropometric and clinical data collected 5 years apart.

	Male			Female		
	1^st^ time point	2^nd^ time point	*P* value	1^st^ time point	2^nd^ time point	*P* value
No. subjects	25			49		
Age (year)	56.0 ± 8.9	61.2 ± 9.0	**< 0.001**	50.4 ±12.4	55.1 ±12.5	**< 0.001**
Range of age	38–75	43–80		30–78	36–83	
Weight (kg)	56.3 ± 9.1	58.4 ± 10.7	**0.010**	55.3 ± 9.8	57.9 ± 10.0	**< 0.001**
Height (cm)	160.3 ± 4.3	160.3 ± 4.4	0.861	151.0 ± 5.5	151.0 ± 5.5	0.631
BMI (kg/m^2^)	21.9 ± 3.1	22.7 ± 3.6	**0.009**	24.3 ± 3.7	25.3 ± 3.9	**< 0.001**
WC (cm)	79.5 ± 10.1	82.1 ± 10.1	**0.037**	82.4 ± 9.5	86.6 ± 10.7	**< 0.001**
SBP (mmHg)	124.8 ± 12.7	124.3 ± 12.0	0.829	125.3 ±13.3	124.1 ± 18.8	0.394
DBP (mmHg)	79.6 ± 8.3	80.8 ± 9.2	0.208	78.9 ± 9.6	77.2 ± 10.7	0.143
HR (bpm)	71.2 ± 8.7	77.2 ± 12.4	**0.049**	79.5 ± 11.4	82.7 ± 13.0	0.056
Blood glucose (mg/dL)	121.1 ± 28.3	143.9 ± 51.3	**0.044**	118.0 ± 39.2	160.3 ± 83.6	**0.002**
HbA1c (%)	5.1 ± 0.5	5.5 ± 0.8	0.172	5.5 ± 0.9	7.1 ± 2.5	**0.001**

Data are mean ± SD or number

Comparison of data collected first (in 2007 and 2008) with data collected second (in 2012 and 2013), using the Wilcoxon signed-rank test for paired comparisons.

Significant *P* values (<0.05) are in bold.

WC; Waist circumference, SBP; Systolic blood pressure, DBP; Diastolic blood pressure, HR; Heart rate.

### Relationship between *APOE* ε4 carrier/non-carrier status and examination characteristics

**Fig 1:**
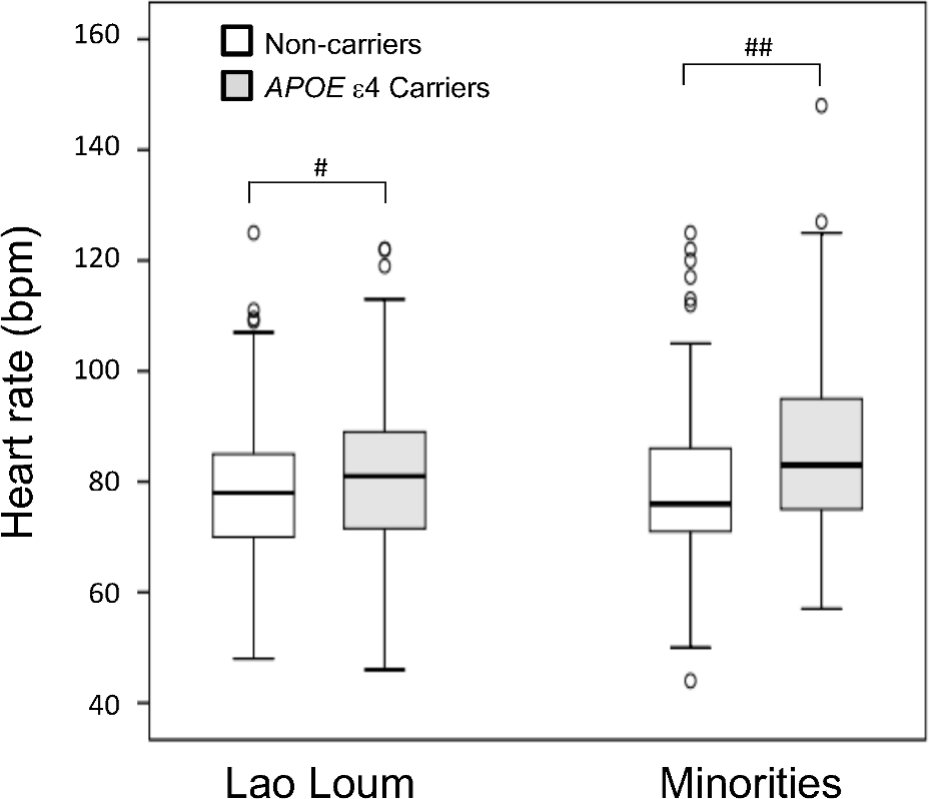
Comparison of *APOE* ε4 Carriers with non-carriers. Statistical analysis was performed using the Mann–Whitney U test (#; *P* < 0.05, ##; *P* < 0.01, significant difference relative to non-carriers). Outliers are represented by open circles (values between 1.5 and 3 times greater than the interquartile range).

Supporting information S1 Table shows the effects of *APOE* ε4 on anthropometric characteristics, blood pressure, and blood glucose in each ethnic group. Interestingly, resting HR was significantly higher in the *APOE* ε4 carrier group than in non-carriers in both Lao Loum and minorities (Fig 1). Body weight, height, BMI, WC, SBP, DBP, blood glucose, and HbA1c were not significantly different in either group. To determine the effects of age, sex, ethnicity, and *APOE* ε4 on anthropometric characteristics, simultaneous multiple regression analyses were performed as shown in Table 5. We used the anthropometric or clinical data points as the dependent variables, and age, sex, ethnicity, and *APOE* ε4 as independent variables. After adjustment for other independent variables, HR was significantly associated with *APOE* ε4. Although the association was weak, *APOE* ε4 significantly increased HR, but did not affect other dependent variables.

**Table 5:** Simultaneous multiple regression analyses.

Dependent variable	Regression coefficient	Constant	Independent variables				Model summary (ANOVA)
			Age	Sex	Ethnicity	*APOE* ε4	
Weight	Unstandardized B	89.081	-0.150	-5.442	-14.624	-0.807	R^2^ = 0.310
	Standardized Beta		-0.189	-0.215	-0.541	-0.032	F(4, 571) = 64.08
	*P* value	**0.000**	**0.000**	**0.000**	**0.000**	0.376	***P* = 0.000**
	Partial correlation coefficient		-0.206	-0.249	-0.508	-0.037	
Height	Unstandardized B	181.437	-0.110	-10.106	-4.773	0.750	R^2^ = 0.475
	Standardized Beta		-0.223	-0.639	-0.283	0.047	F(4, 570) = 128.73
	*P* value	**0.000**	**0.000**	**0.000**	**0.000**	0.132	***P* = 0.000**
	Partial correlation coefficient		-0.273	-0.659	-0.332	0.063	
BMI	Unstandardized B	29.708	-0.033	0.716	-4.958	-0.508	R^2^ = 0.243
	Standardized Beta		-0.116	0.078	-0.504	-0.055	F(4, 570) = 45.79
	*P* value	**0.000**	**0.003**	**0.034**	**0.000**	0.144	***P* = 0.000**
	Partial correlation coefficient		-0.122	0.088	-0.464	-0.061	
WC	Unstandardized B	107.985	0.089	0.150	-28.923	-0.065	R^2^ = 0.469
	Standardized Beta		0.072	0.004	-0.656	-0.002	F(4, 552) = 122.09
	*P* value	**0.000**	**0.032**	0.905	**0.000**	0.960	***P* = 0.000**
	Partial correlation coefficient		0.091	0.005	-0.635	-0.002	
SBP	Unstandardized B	92.400	0.401	1.252	8.562	1.408	R^2^ = 0.119
	Standardized Beta		0.357	0.035	0.219	0.039	F(4, 564) = 19.11
	*P* value	**0.000**	**0.000**	0.384	**0.000**	0.341	***P* = 0.000**
	Partial correlation coefficient		0.332	0.037	0.207	0.040	
DBP	Unstandardized B	68.320	0.037	0.351	5.899	-0.083	R^2^ = 0.057
	Standardized Beta		0.055	0.016	0.253	-0.004	F(4, 564) = 8.49
	*P* value	**0.000**	0.217	0.693	**0.000**	0.928	***P* = 0.000**
	Partial correlation coefficient		0.052	0.017	0.230	-0.004	
HR	Unstandardized B	75.706	-0.083	5.128	-1.731	3.510	R^2^ = 0.069
	Standardized Beta		-0.099	0.191	-0.060	0.131	F(4, 564) = 10.45
	*P* value	**0.000**	**0.024**	**0.000**	0.183	**0.002**	***P* = 0.000**
	Partial correlation coefficient		-0.095	0.193	-0.056	0.130	
Blood glucose	Unstandardized B	130.998	0.712	-15.341	-10.728	-3.215	R^2^ = 0.068
	Standardized Beta		0.175	-0.118	-0.077	-0.025	F(4, 563) = 10.28
	*P* value	**0.000**	**0.000**	**0.004**	0.087	0.559	***P* = 0.000**
	Partial correlation coefficient		0.165	-0.121	-0.072	-0.025	

Significant *P* values (<0.05) are in bold

Sex; male(1) and female(2)

Ethnicity; Lao Loum(1) and minorities(2)

*APOE* ε4; absence of *APOE* ε4 (0) and presence of *APOE* ε4 (1)

WC; Waist circumference, SBP; Systolic blood pressure, DBP; Diastolic blood pressure, HR; Heart rate.

## Discussion

In this study, we analyzed the *APOE* allele distribution in Laos. The frequency of *APOE* ε4 was higher among minorities than Lao Loum. This study raised some important public health issues regarding obesity, hypertension, and diabetes in Laos. Trends for BMI, WC, SBP, DBP, blood glucose, and HbA1c in Lao Loum females across age groups were mostly highly significant. Comparisons of data regarding anthropometric and clinical characteristics collected 5 years apart revealed that body weight, BMI, WC, and blood glucose increased significantly in Lao Loum of both sexes. On the other hand, in female minorities across age groups, BMI and WC were on a significant declining trends, and in both sexes of minorities SBP and DBP were no significant different. About trend for body weight across age groups, tendency to decrease with age was observed in Lao Loum both sexes and female minorities. However, the highest body weight was observed in middle age of majority, but not in minorities. These results suggested that Lao Loum experience obesity, elevated blood pressure, and higher blood glucose in middle age by both age-related change and lifestyle change, such as high calorie foods and motorization. Contrarily, in minorities only the blood glucose level of males increased with age among people in their 20s to 40s groups, suggesting that blood glucose level may be strongly affected by age. On the other hand, the change of living environment and lifestyle may promote a rise in obesity and blood pressure. Economic growth in the big cities of Laos has been remarkably fast. In Cambodia, a neighboring country of Laos, diabetes is considerably more frequent than was previously expected[34]. Diabetes is not yet one of the top 10 diseases in Laos, but mortality due to this disease is high (2% of 46,000 annual deaths)[35]. Based on its increasing population and rapid economic growth, Asia is considered to represent a modern diabetes pandemic[34]. In contrast to Lao Loum, increases of BMI, WC, SBP and DBP were barely observed among ethnic minorities as mentioned above. Additionally, minorities had smaller physiques than Lao Loum. One reason for this difference is that the minorities were still in economic poverty and had a high malarial infection rate relative to Lao Loum in urban areas. There is an economic and health disparity between the majority and minorities in Laos[1]. Before the intervention by the government along with WHO and other development partners in 2001, the minority village was in a malaria-endemic area[1, 36]; however, the numbers of admissions and deaths from malaria have decreased over the past decade[36]. Older people born before the intervention might have been infected with malaria several times during their lifespan. In younger age groups of minority females and Lao Loum of both sexes, height was greater than in older age groups (Table 3 and S1 Fig), presumably due to economic growth and the medical service strategy against infectious diseases. Although people in poverty-stricken areas are thought to be at lower risk of non-communicable diseases, minorities in Laos may follow the same path as the Lao Loum in the near future.

*APOE* allele distribution differs according to ethnicity and region. Corbo *et al*. reported that some Africans have high frequencies of *APOE* ε4 (Pygmies 40.7%, Khoi San 37.0%). Among Europeans, the prevalence is higher in Northern Europe (Swedish 20.6%, Finnish 20.8%) and lower in Southern Europe (Italians 9.1%, Greeks 6.8%)[37]. Among Asians the prevalence is low (Chinese 7.1%, Japanese 10.1%)[37]. In this study, we found that the *APOE* ε4 allele frequencies of minorities (Talieng 24.3%, Alak 40.1%) were higher than those of Lao Loum (12.6%). Among Asians, these two minorities have a very high occurrence of the *APOE* ε4 allele. The allele is also very prevalent in Malay and American aboriginals (Malay 24.0%, Inuit 21.4%)[37]. Of the three common alleles of *APOE*, the so-called ‘thrifty’ ε4 allele is an ancestral allele that has been selected because it protects against some infectious diseases and increases cholesterol[38, 39]; thus, *APOE* ε4 may improve survival in populations experiencing food scarcity or poverty[38]. Jofre-Monseny *et al*. also reported that *APOE* ε4 plays a role in protecting against certain infectious diseases, and may have provided an initial evolutionary advantage related to pathogen resistance in developing countries[38]. Economic expansion, gradual adoption of a Western lifestyle (including a high-fat diet), low levels of physical activity, and long life expectancy have resulted in a shift from infectious diseases to non-communicable diseases. The elevation in the rates of non-communicable diseases such as hypertension and diabetes is likely to increase the burden of dementia, including AD [14]. Bang *et al*. showed that the frequency of *APOE* ε4 was significantly higher in AD patients than in controls in Caucasians, Southern Europeans, and East Asians[40]. Farrer *et al*. suggested that although *APOE* ε4 represents a major risk factor for AD across all ages between 40 and 90 years in both sexes, the attenuated effect of *APOE* ε4 in some ethnicities was caused by small sample size, population heterogeneity, or other factors[10]. The effect of ethnicity on the association between *APOE* genotype and AD remains unclear; consequently, further investigations are necessary.

In a statistical analysis of anthropometric and clinical characteristics between *APOE* ε4 carriers and non-carriers, only heart rate was significantly higher in *APOE* ε4 carriers than in non-carriers in both Lao Loum and minorities. Several reports demonstrated that *APOE* ε4 is associated with AD[40], cognitive decline[21], depressive symptoms[22], stroke[23, 24], hypertension[25, 26], coronary heart disease[24, 26], cardiovascular[27, 28], and diabetes[29, 30]. Very few studies have addressed the relationship between *APOE* ε4 and heart rate. Cheng *et al*. reported that heart rate variability is significantly associated with reduced physiological complexity in the presence of the *APOE* ε4 allele, relative to non-carriers[41]. Elevated resting HR is a risk factor for cardiovascular disease in healthy males and females[42, 43] and is also associated with elevated risk for the development of insulin resistance[44]. Scuteri *et al*. described higher levels of pulse wave velocity in relation to dementia [45, 46]. Based on the observations described above, elevated HR may be a conventional index for cardiovascular disease, diabetes and dementia in *APOE* ε4 carriers.

Our study is the first to report population data on *APOE* ε4 allelic frequencies in Laos, although the sample size is small. The very high occurrence of the *APOE* ε4 allele may increase susceptibility to several non-communicable diseases in two minorities of Laos. Additionally, the prevalence of non-communicable diseases is rapidly increasing in the Lao Loum majority. In the primary government hospital in Vientiane, one psychiatrist saw only two patients with impaired memory in more than 20 years (personal communication). Dementia, including AD, is not yet a serious public health problem in Laos. Caution against genetic prediction and testing using *APOE* genotyping for AD is recommended [47], particularly to guard against genetic determinism, especially in ethnic minority groups. However, our findings at least suggest a genetic predisposition of the Lao population to non-communicable diseases. Our study provides informative data regarding ethnic differences in the association between *APOE* and non-communicable diseases or dementia. Given the small sample size for the groups in this study and the fact that few subjects participated twice in measures over the 5-years period, it is possible that our findings may have been affected by selection bias or non-participation bias in the measures. Therefore, additional studies that include larger sample sizes are necessary to address these limitations. Further investigations in larger Laotian ethnic minority groups are necessary to confirm these findings. In the meantime, however, clinicians and researchers should consider prevention strategies that target cardiovascular, diabetes and dementia risk factors.

## Supporting Information

S1 Fig Trends in age groups of Lao Loum and minorities. Statistical analysis was performed using the Jonkheere–Terpstra test for trends across age groups. Significant differences across all age groups are indicated by an arrow, and significant difference between the 20s and 40s alone is indicated by a dashed arrow (#; *P* < 0.05, ##; *P* < 0.01). Outliers are represented by open circles (values between 1.5 and 3 times greater than the interquartile range) or asterisks (more than 3 times greater). WC: waist circumference; SBP: systolic blood pressure; DBP: diastolic blood pressure; HR: heart rate.
(TIF)

S1 Table Demographic factors, anthropometric and clinical characteristics among *APOE* ε4 carriers and non-carriers. (TIF)
