# Supplementary figures and images for: APOE Genotype in the Ethnic Majority and Minority Groups of Laos and the Implications for Non-Communicable Diseases
Source: PLoS One. 2016 May 11;11(5):e0155072. doi: 10.1371/journal.pone.0155072 (PMC4863969; doi:10.1371/journal.pone.0155072)

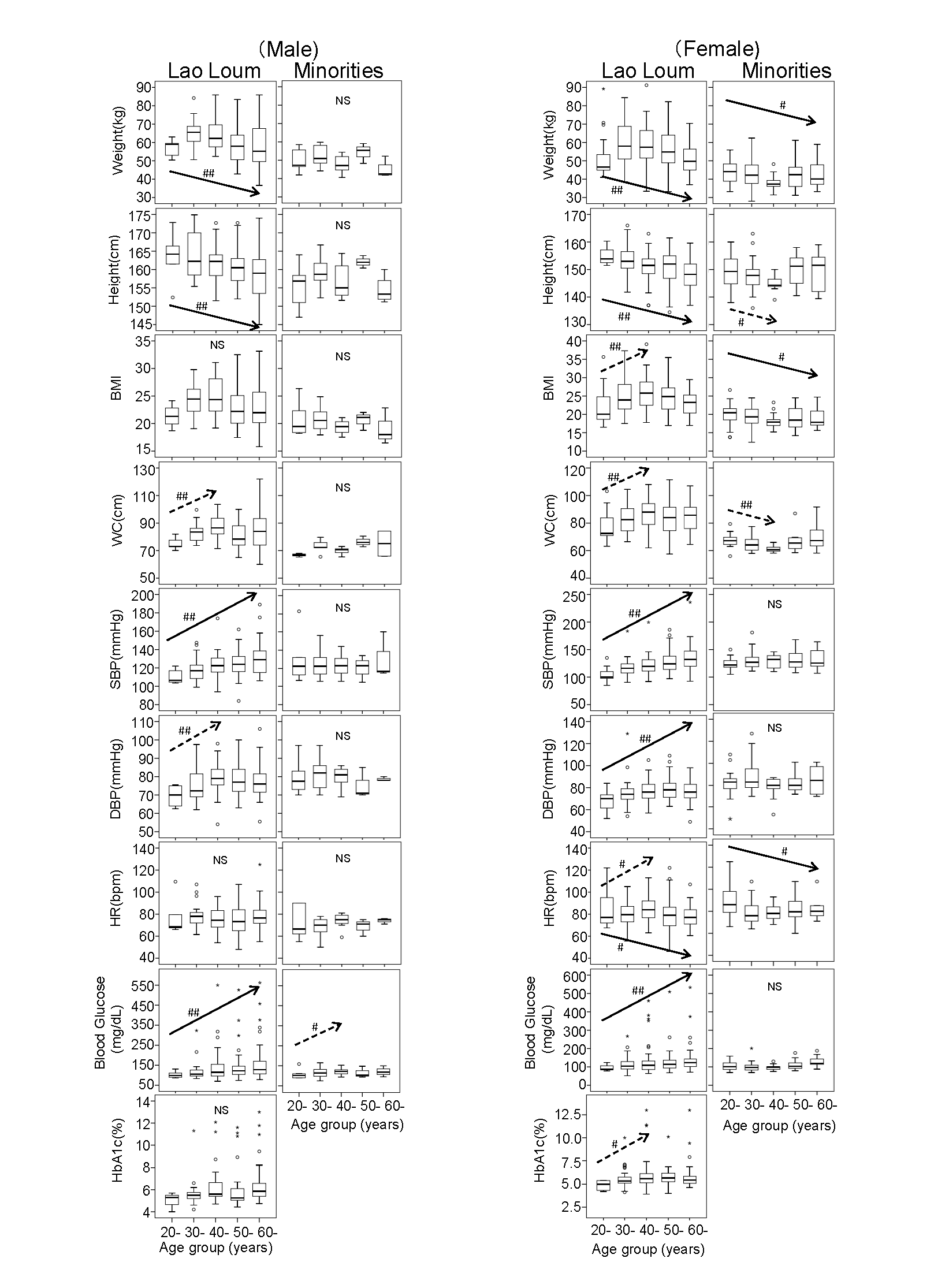

Supplement: S1 Fig — Statistical analysis was performed using the Jonkheere–Terpstra test for trends across age groups. Significant differences across all age groups are indicated by an arrow, and significant difference between the 20s and 40s alone is indicated by a dashed arrow (#; P < 0.05, ##; P < 0.01). Outliers are represented by open circles (values between 1.5 and 3 times greater than the interquartile range) or asterisks (more than 3 times greater). WC: waist circumference; SBP: systolic blood pressure; DBP: diastolic blood pressure; HR: heart rate. (TIF) [file pone.0155072.s001.tif]
